# Introduction to the Special Issue on Working With High‐Conflict Families in Custody Contexts: A Call to Action

**DOI:** 10.1111/jmft.70087

**Published:** 2025-11-05

**Authors:** William F. Northey, Jeff Chang, Erin Guyette

**Affiliations:** ^1^ Bellefonte Center for Children and Families Wilmington Delaware USA; ^2^ Independent Practice, Director of Clinical Training, MindKey Health Victoria British Columbia Canada; ^3^ Guyette Family Guidance, PLLC Maple Grove Minnesota USA

## Abstract

Families presenting for therapy after a divorce or separation who are in high‐conflict present marriage and family therapists (MFTs) with some of the most complex and ethically challenging cases. Most co‐parents settle disagreements amicably; however, 8–15% engage in conflict for years post‐separation. Therapists must navigate overlapping clinical, legal, regulatory, and systemic domains—tasks for which most MFTs receive little training. This special issue of the *Journal of Marital and Family Therapy* addresses the urgent need for therapist competency and interdisciplinary collaboration with families in high conflict. Drawing on systemic and structural approaches, the featured articles examine factors that shape co‐parenting quality, strategies for introducing new partners, reunification models, and the lived experiences of parents facing parent–child contact problems. Integrating research with practice‐informed insights, this issue advocates for therapy with families in high‐conflict as a clinical specialization and calls for advanced training, certification, and stronger collaboration among professionals.

## 1 Rationale for This Special Issue

The impetus for this special issue arose from our recognition that, despite the systemic orientation and clinical competencies of marriage and family therapists (MFTs), relatively few provide services to families entrenched in high‐conflict dynamics postdivorce. In many clinical settings, there is no established framework to adequately address the complexities of these cases. Many of these cases come into clinics that do not have a process to meet the needs of these cases. While most divorcing parents separate with minimal conflict, approximately 8 and 15% of parents remain engaged in ongoing legal battles, emotional entanglement, or prolonged involvement of children in disputes for several years post‐separation (Kelly 2000). These families need the support of well‐prepared therapists, and therapists, in turn, must feel confident to meet the unique challenges inherent in this study.

A major concern for the field is the lack of educational opportunities that prepare therapists to competently service postdivorce families in high conflict. As MFTs who work in this context, we acknowledge and appreciate the challenges these families present to therapists, including testifying in family courts, working with attorneys and custody evaluators, navigating behavioral health and substance use disorders, and responding to complaints filed with regulatory boards. Navigating these areas is not generally taught in MFT training programs. Because some parents can be litigious, clinical educators, particularly those teaching ethics, warn students to avoid working with such families altogether. This tendency contributes to a cascade effect: as MFTs avoid this study, they have limited opportunities to develop competence, are more likely to decline such cases, and inadvertently transmit this avoidance to the next generation of therapists.

A common concern therapists have is the fear of complaints filed with regulatory boards due to litigious parents. This concern is understandable, as licensure depends on adherence to professional standards, and this is how we keep our careers. However, therapists can ethically serve families in high conflict just as they would any other population. Avoiding this study out of fear of complaints is unnecessary, as the ethical principles guiding our practice remain the same regardless of the client population. Although no specific data exists on how many complaints are specifically related to child custody issues, data from Bow and Quinnell (2001) found that less than 1% of complaints resulted in formal findings. A similar pattern emerges from the Florida Department of Health, Division of Medical Quality Assurance (2024), which found that there were over 50,000 complaints in 2023–2024, but only 5000 resulted in an investigation. Anecdotally, a senior psychologist working for a Canadian regulatory board estimated that one‐third of their time is spent on complaints originating in contested parenting matters. Given the litigious nature of some parents involved in high‐conflict conflict, it is not surprising that complaints to regulatory boards are sometimes used as a litigation tactic in family law proceedings. We share this not to scare MFTs away from this study, but to recognize the potential opportunity for MFTs by virtue of our systemic perspective. To that end, we have curated this special issue to invite both training programs and clinicians to consider providing services to these families. Hopefully, with education, this can reduce existing fears of working with these families, as there is a high need for mental health support for these families.

Generally, training programs in MFT discuss systems thinking and theoretical approaches, which provide an excellent foundation to start the work with high‐conflict families. However, this is not enough due to the need for knowledge about legal proceedings and potential legal involvement. Additional competence in mediation, therapeutic boundaries, conflict‐resolution, family law, collaboration with legal professionals, and navigation of ethical challenges is imperative to intervene safely, effectively, and ethically with these families (Northey and Gehart 2020). Because of our systemic approach, MFTs are particularly qualified to provide services including co‐parenting counseling, reunification/relationship reconciliation therapy, parenting coordination, and co‐parenting and custody evaluations with appropriate training and consultation (Rautio et al. 2025).

## 2 Overview of Special Issue Topic

The escalation of marital separations and divorces that are high‐conflict has intensified the demand for MFTs equipped to navigate the complex interplay of relational dynamics, psychological trauma, and legal mandates. These families often find themselves entangled in contentious custody disputes, strained parent‐child relationships, and a revolving door of court involvement that places therapists at the heart of emotionally charged, ethically ambiguous terrain. Despite this reality, the field of family therapy lacks a cohesive, empirically grounded framework for addressing the unique needs of these families and adequately preparing therapists to work with them. This special section of the *Journal of Marital and Family Therapy* aims to begin to bridge that gap by presenting four original articles that illuminate the contours of therapeutic work with high‐conflict families and offer innovative, practice‐informed solutions.

Decades of family systems theory have emphasized the recursive nature of relational patterns, but as Guyette (2024) reveals, therapists often feel ill‐prepared, emotionally drained, and unsupported in the face of the intense relational dynamics and challenges posed by high‐conflict custody cases. There is little research on the perspectives of therapists in this study, so Guyette (2024) completed an exploratory study. From the findings, many MFTs report being “thrown in” to these cases without adequate supervision, training, or guidelines, contributing to fear, professional burnout, and inconsistent care (Guyette 2024). These qualitative findings underscore the critical need for standardized training, certification processes, and interdisciplinary collaboration—elements echoed in the research articles presented in this volume.

This special issue aims to explore not only the therapeutic frameworks that can be applied directly with clients, but also the critical importance of understanding the broader contexts in which families operate. Effective intervention requires therapists to engage in meaningful collaboration and communication with other professionals involved in the family's various systems, such as legal, educational, and social services. By adopting a holistic, interdisciplinary approach, therapists can better address the multifaceted challenges these families face and support more comprehensive, coordinated care. This process engages core competencies grounded in a systemic orientation and collaborative practice, drawing upon MFTs expertise in analyzing relational processes and implementing coordinated interventions across interconnected systems.

## 3 Summary of Articles

Fitzgerald et al. (2025) explore how the stated reasons for divorce by the separating parents—specifically family violence and parenting differences—impact the coparenting relationship and, through it, children's emotional and behavioral outcomes. Using a sophisticated *common fate model*, they highlight how shared and individual partner perceptions shape the postdivorce family environment. This study exemplifies how systemic conceptualizations can better illuminate the embeddedness of conflict than explanations based on individual pathology. This suggests that therapeutic interventions must be attuned to pre‐separation dynamics to address post‐separation challenges.

Kang and Nielson (2025) examine a sensitive but common issue in postdivorce adjustment: how parents communicate new romantic relationships to their children. They offer clinical guidance grounded in family resilience theory, emphasizing timing, developmental sensitivity, and parental self‐awareness. When parents are highly conflictual, introducing new partners can escalate existing tensions. This offering provides a clinical roadmap for therapists supporting co‐parents and children through transitions that are often laden with loyalty conflicts and boundary ambiguities.

Singh and Mader (2025) present an integrative, single‐therapist framework for reunification therapy, tailored to the treatment of parent‐child contact problems (PCCPs). Informed by systemic, attachment, and cognitive‐behavioral traditions, the proposed model offers a flexible, process‐oriented structure for therapists navigating the treacherous waters of *alienation*, *estrangement, and resist/refuse dynamics*. This contribution addresses the field's current fragmentation, providing clinicians with a coherent treatment map that balances the court's expectations with therapeutic integrity.

Guyette and Harris (2025), using a qualitative approach, focus on parents with strained relationships postdivorce and reveal the often‐invisible emotional toll of PCCPs. Parents describe feeling misunderstood by professionals, disempowered by legal constraints, and desperate for therapeutic alliances that are both empathetic and effective. These findings echo those of Guyette (2024), where therapists, too, reported feeling overwhelmed, inadequately trained, and caught between competing professional demands. Both bodies of research call for specialized, trauma‐informed, and legally literate approaches to care.

Collectively, the articles in this special issue advocate for the formalization of high‐conflict family therapy as a clinical specialization. As Guyette (2024) suggests, the creation of advanced training programs, certification pathways, therapy liaison roles, and structured consultation networks would equip therapists with the tools necessary to meet these challenges with confidence and competence. Moreover, coordinated training for legal professionals could foster greater understanding across disciplines and promote more holistic care for families entangled in custody disputes.

## 4 Strengthening Clinical Training in Therapy With Families in High‐Conflict

MFTs typically receive little to no formal education on the legal system or its implications for clinical practice and family functioning. Graduate curricula generally focus on systems theory, assessment, and treatment, with limited coverage of the intersection of mental health and family law. Consequently, many MFTs lack confidence and competence to navigate situations involving family court or child custody disputes. This knowledge gap often leads to confusion about roles, ethics, and responsibilities, especially concerning confidentiality, documentation, and responding to subpoenas for court testimony. Without a clear grasp of court procedures, therapists risk compromising therapeutic relationships or inadvertently placing themselves and their clients in jeopardy.

Given the constraints of typical MFT programs, there are few opportunities to practice systemic interventions with specialized clinical populations such as families experiencing high‐conflict divorce and parenting. Many MFTs recall educators—particularly ethics instructors who do not practice in this area—reflexively warning against involvement in these cases, citing legal risk and emotional intensity. The adversarial legal system in the United States and other common‐law countries often exacerbates these fears, and supervisors or faculty reinforce knee‐jerk reluctance to engage with this population (Chang and Vath 2024).

MFTs often encounter a range of ethical challenges when their clinical role intersects with legal demands: maintaining client confidentiality while responding to court orders, subpoenas, or requests for testimony; the inherent conflict between the roles of treating therapist and expert witness; acting in the best interest of the client, however that is operationalized; and the pull (either from a client or from our sense of urgency) to align with one client more than another. As such, navigating the legal system requires a strong ethical foundation, knowledge of legal principles, and ideally, specialized training in forensic behavioral health.

To begin changing the training approach, integration of theoretical models should be considered to provide structure for working with families in high conflict. For example, an approach to conceptualizing family interactions, such as the IPscope (“IP” standing for *interpersonal patterns*) incorporates individual, parental, structural, community (Tomm et al. 2014), and legal (Chang 2023) factors, situating family dynamics within broader ecological and legal contexts. Seeing conflict‐generating behaviors as embedded within interpersonal patterns as opposed to merely individual pathology can help MFTs maintain a systemic view and reduce cognitive dissonance when faced with “he said/she said” scenarios. This can help MFTs conceptualize cases, prioritize interventions, and remain grounded amid the emotional and procedural turbulence of court‐involved work. The IPscope is but one example of an approach that can help MFTs' training and supervision (Chang et al. 2020) can improve clinical clarity, reduce therapeutic drift, and strengthen accountability.

Northey and Chang (2018) emphasized the necessity of a developmental and systems‐based approach. They highlighted the prevalence of emotionally reactive behaviors, hostile attribution biases, and exaggerated parenting disputes in high‐conflict divorces—issues that therapists must be equipped to navigate with both clarity and neutrality. Their recommendations include structured interventions across pre‐separation, post‐separation/pre‐divorce, and postdivorce phases, with a focus on sustaining the therapeutic relationship, engaging the parental subsystem, ensuring therapist safety, and maintaining a long‐term systemic perspective. These insights align closely with the articles in this issue and provide a practical, clinical scaffolding for the types of interventions needed in court‐involved family therapy.

The importance of formalized training, and possibly certification, is echoed in the work of Northey (2009), who has long argued for the legitimization of marriage and family therapy as a profession grounded in evidence‐based practice and professional standards. Northey's contributions underscore the necessity of systemic competencies and the creation of structured education and credentialing pathways that align with the complex demands of contemporary family therapy. His insights offer a framework for understanding how professionalization can serve both MFTs and the high‐conflict families they serve.

The development of a credential or certification for MFTs and other behavioral health professionals to work with high‐conflict families will take a concerted effort by numerous stakeholders to achieve. In the meantime, we believe it is important to highlight the areas of competence critical to assist MFTs and other therapy professionals in providing services to families in high conflict (Northey and Gehart 2020).

## 5 Potential Competency Areas of Training

First, and possibly most importantly, is the application of systemic and relational competencies. Systemic assessments, like genograms, structural maps, and ecomaps, can be extremely useful in recognizing the patterns that maintain the conflictual patterns in these families. Structural therapy provides a solid foundation for exploring the issues and challenges presented by high‐conflict families. Narrative, strategic, and other post‐modern therapy approaches that highlight and embrace the “parallel realities” that are endemic can assist MFTs in accepting the “both/and” and balancing both the real and presumed alliances and perspectives that are part and parcel of the therapy with highly conflictual co‐parents. In fact, Karl Tomm (McIntosh et al. 2023) has suggested that it might be more useful to think of these families and parents as “high discrepancy” rather than “high conflict.” It almost goes without saying that effective systemic reframes, whether benign or benevolent, are crucial to success with these client situations. These families are the epitome of being stuck, and helping them get unstuck can be a challenge.

The ability to manage conflict and help clients develop effective communication are critical to this study. Sometimes clients lack the skills to communicate effectively, but more likely, their pain and anger fuel aggressive and hurtful “zingers.” MFTs can utilize structured dialogs, time‐outs, shuttle diplomacy (talking with parents individually to decrease reactivity), and even directly managing interactions (“shooshing”). At these times, MFTs may be outside of their comfort zone of being supportive and curious to feeling more like the chairperson of a volatile meeting. Boundary management, clarifying how much say each parent will have in the other's home, is also quite helpful, in addition to clear expectations about appropriate behavior during sessions or when communicating via text messages. For example, OurFamilyWizard (OFW), an application that allows for text and audio communication with a shared calendar, can be effective, especially when the MFT teaches application of resources to the coparents. OFW offers many features, such as an AI Tone Meter that can alert clients to negative messages and write the message for the clients (https://www.ourfamilywizard.com/).

When communication is especially problematic, it can be useful to provide parents with direct guidance, verbal or written. An MFT can even offer to review and edit draft messages to the other parent, helping prevent or reduce negative interactional patterns. As noted above, applications like OFW, Talking Parents (https://talkingparents.com/), and Large Language Models (e.g., ChatGPT, Claude) can be very helpful in assisting clients to reduce their negativity and enhance their communication. It is often necessary, if emotional reactivity is difficult to manage in a conjoint session, to have individual sessions with parents to examine the factors contributing to the conflict. Sometimes a referral to an individual therapist is necessary.

Knowledge of how the family court system interfaces with the clinical system is essential. MFTs can become involved with families throughout the separation, divorce, and postdivorce process. Understanding the types of custody there are (e.g., legal, residential), residential custody arrangements, the meaning of court orders, and recognition of different roles MFTs play (e.g., co‐parenting counselor, R/RRT, parenting coordinator, mediator, therapist to a particular subsystem, or custody or coparent evaluator) in these families is imperative. Engagement with high‐conflict parents will inevitably lead to being subpoenaed and testifying in court, either as a fact or expert witness. Consequently, being aware of the role you have been asked to fill is essential, as well as documenting in a way that will stand up to legal scrutiny if asked to produce documents for the court. It is also necessary to understand that the author of any document, whether it is a brief letter describing therapy progress or a comprehensive child custody evaluation, is subject to examination and cross‐examination. Generic informed consent forms are generally inadequate for this study, given the type of services and roles being served. Avoiding dual roles (e.g., custody evaluator and therapist) is both practically and ethically necessary. MFTs must also maintain clear boundaries with the legal system (i.e., lawyers, judges, Guardian Ad Litem) to work effectively, as they are not often familiar with our ethical codes.

This study is wrought with ethical and legal challenges (Association of Family and Conciliation Courts 2011; Chang and Vath 2024). Navigating polarized narratives, an adversarial family court system, while looking out for the best interests of the children involved, is exacting and specifying all of the issues is beyond the scope of this introduction. It is noteworthy that to avoid unfounded claims of ethical violations, MFTs should have clear informed consent documents describing the parameters of services, ownership of personal health information, and the parameters for release of information, and obtaining and reviewing all legal documents. Balancing and maintaining appropriate clinical boundaries is imperative and should be reflected in case documentation. Documentation that is focused on “just the facts,” that is, observations and direct quotes, will serve MFTs well should they be subpoenaed or when clients ask to review their records. Perhaps most importantly, taking care of yourself, getting support, supervision, and consultation, and expanding competence is fundamental to maintaining an effective and ethical practice.

There are other competencies common to many other areas of difficult clinical presentations. Risk assessment and safety planning to screen for child abuse, domestic violence, suicidal ideation, substance use, and behavioral health disorders are required for best practice. Similarly, we must be culturally competent, particularly with respect to parenting values and practices, which often undergird parenting conflicts. As MFTs, our own values, beliefs, and expectations influence our ability to intervene effectively with high‐conflict co‐parents. Given the level of conflict in these families, self‐care and clear boundaries are crucial. Monitoring countertransference, compassion fatigue, vicarious trauma, and burnout is necessary to remain psychologically healthy when working with complex issues and systems.

Ultimately, working with high‐conflict co‐parents demands a blend of systemic expertise, legal literacy, ethical resilience, and personal sustainability—competencies that should be fostered intentionally within MFT education and professional development.

## 6 Future Directions and Challenges

This special issue arrives at a pivotal moment to advance the field. As high‐conflict divorce becomes more common and as family therapists become increasingly involved in family court, the field must evolve. We must develop evidence‐informed, ethically robust, and systemically grounded models of care that recognize the multi‐dimensional realities of families engaged in high‐conflict custody disputes. In doing so, we can better support the well‐being of children by mitigating the strained parent–child bonds and ideally promoting the long‐term health of the entire family postdivorce. As outlined in this special issue, MFTs possess foundational knowledge, but working effectively with families in high‐conflict postdivorce requires specialized legal and clinical knowledge, advanced systemic tools, strong risk management skills, and ongoing support to promote the well‐being of children affected by high‐conflict dynamics. There are many MFTs, including those who contributed to the special issue, who have developed the competency. However, no explicit path to developing said skills currently exists. There are sister organizations to the AAMFT, like the Association of Family and Conciliation Courts (https://www.afccnet.org/), Family Mediation Canada (https://fmc.ca/), and the Academy of Professional Family Mediators (https://apfmnet.org/), who provide many excellent resources and professional development activities to develop the requisite competence. The AAMFT could facilitate the advancement of competence in working with high‐conflict coparenting by creating tracks at their conferences or supporting a topical interest group focused on divorce and co‐parenting. Exploring the development of a certification program might also be helpful so these therapists are recognizable to other therapists, attorneys, mediators, judicial officers, and other professionals who work with these families. Further, online training and practice guidelines focused on working with this population would serve not only the membership generally but also contribute to the expansion of services for families. Supervision and mentor programs might also bode well for developing a cadre of MFTs who can help families in conflict postdivorce. The guidance of seasoned professionals can help spread the knowledge for generations to come.

Regarding evidence‐based practice, there are numerous challenges that exist because of the relatively low frequency of research and the need for longitudinal studies. Furthermore, studying high‐conflict co‐parenting is methodologically and ethically complex because of legal entanglements, participant mistrust, consent barriers, and the volatile nature of these family systems. These challenges necessitate specialized recruitment strategies, rigorous ethical safeguards, and researcher support systems. Parents are also likely to be reluctant to engage in research due to the litigious nature of these custody cases. Getting the perspectives of both parents and children creates barriers for practical, ethical, and legal reasons. The use of qualitative methods, like Guyette (2024), could provide insights into what therapeutic interventions could be most effective. A collaborative research practice approach, where multiple clinicians collect data on the clients with whom they work, could be a potential solution to the challenges of doing research on high‐conflict clients. Although it would probably be easier to do with the ubiquity of electronic health records, there would likely need to be a considerable investment in creating such an endeavor.

Despite the challenges ahead for advancing effective interventions with high‐conflict coparents, this area of practice presents opportunities for MFTs to expand their practices (Chang 2019). In fact, we would argue that MFTs, with our systemic and social justice orientations, are better positioned than other behavioral health professions. In fact, clinical approaches that are dominated by individualistic thinking can exacerbate conflict (Chang and Vath 2024). Finally, to put it simply, children of high‐conflict divorcing parents need us! The evolution described in this special issue holds promise for transforming the therapeutic landscape, ensuring that therapists are equipped to meet these challenges with competence, compassion, and clarity, thereby fostering healthier outcomes for families and communities alike.
